# Aquaporin-1 Expression in Proliferative Vitreoretinopathy and in Epiretinal Membranes

**DOI:** 10.1155/2014/876208

**Published:** 2014-02-04

**Authors:** Elie Motulsky, Dany Salik, Xavier Janssens, Bart Pion, Rebecca Dufrane, Florence Chaput, Nargis Bolaky, Françoise Gregoire, Laure Caspers, Jason Perret, François Willermain, Christine Delporte

**Affiliations:** ^1^Laboratory of Pathophysiological and Nutritional Biochemistry, Université Libre de Bruxelles, Blg G/E, CP 611, 808 Route de Lennik, 1070 Brussels, Belgium; ^2^Department of Ophthalmology, CHU Saint-Pierre, Université Libre de Bruxelles, Brussels, Belgium; ^3^Department of Ophthalmology, CHU Brugmann, Brussels, Belgium; ^4^Department of Ophthalmology, Saint-Anne Saint-Rémi Hospital, Brussels, Belgium; ^5^I.R.I.B.H.M., Université Libre de Bruxelles, Brussels, Belgium

## Abstract

*Purpose*. Aquaporin-1 (AQP1) is involved in cell migration and proliferation; therefore, the purpose of the study was to investigate its expression in proliferative vitreoretinopathy (PVR) and epiretinal membranes (ERM). *Methods*. 19 membranes from PVR and ERM were collected following eye surgery. AQP1 mRNA and protein expressions were determined by RT-qPCR and immunofluorescence in the membranes from PVR and ERM. *Results*. AQP1 mRNA and protein were expressed in both PVR and ERM as shown by RT-qPCR and immunofluorescence. AQP1 protein expression was heterogeneous among and between PVR and ERM and colocalized with alpha-smooth muscle actin (**α**SMA) and with glial fibrillary acidic protein (GFAP). There were a higher percentage of cells coexpressing AQP1 and **α**SMA than AQP1 and GFAP. GFAP and **α**SMA did not colocalize. *Conclusion*. Our data show for the first time AQP1 expression in both PVR and ERM. AQP1 is expressed mostly by the **α**SMA-positive cells, presumably myofibroblasts, but also by GFAP-positive cells, assumed to be glial cells. These original findings warrant further functional investigations aiming at studying the potential role of AQP1 in cell migration and proliferation occurring during the development of PVR and ERM.

## 1. Introduction

Proliferative vitreoretinopathy (PVR) and epiretinal membranes (ERM) are both characterized by abnormal migration and proliferation of retinal cells that form contractile membranes [1–6]. In ERM, this process is usually limited to the formation of membranes above the macula that can contract and wrinkle the retina causing patients to complain of visual impairment such as metamorphopsia, micro- or macropsia, blurred vision, and occasionally monocular diplopia [7–9]. Most ERM are idiopathic but can be associated with ocular diseases (e.g., diabetic retinopathy, retinal vein occlusion, postretinal, or cataract surgeries) [10]. In contrast, PVR follows retinal detachment (RD) and/or a traumatic condition [11, 12]. Classically, PVR membranes can develop on both sides of the retina and progress deeper in the vitreous [13, 14]. Both PVR and ERM are made from different cell types including immune, glial, fibroblast-like and RPE cells [15–18].

Aquaporins (AQPs), a gene family of water transmembrane channel proteins, not only ensure transcellular water transport across biological membranes [19] but also have been recently described as implicated in cell migration and proliferation [20, 21]. Several papers have shown that AQP1 is essential for cell migration of different cell types [20, 22–27]. Interestingly, AQP1 expression has been described on several retinal cell types during physiological and pathological conditions [28–33]. As a prerequisite to address the putative functional role of AQP1 in PVR and ERM development, it is first necessary to investigate the expression of AQP1 in PVR and ERM.

## 2. Material and Methods

### 2.1. Membranes from PVR and ERM

PVR and ERM membranes were removed during surgical treatment of patients, in agreement with the appropriate ethical protocol approved by the institutional review boards of CHU Saint-Pierre and Sainte-Anne Saint-Rémi Hospital. The study was conducted according to the tenets of the Declaration of Helsinki. Conventional vitreoretinal surgery was performed by three different surgeons using a two- or three-port system. Size of vitreoretinal instruments was rarely 20 gauge and routinely 23 gauge. Membranes from 19 eyes from 19 patients (7 women and 12 men), with an average age of 59 ± 4 (*n* = 19), were collected (Table 1).

### 2.2. RNA Extraction and cDNA Synthesis

Membranes from PVR and ERM were immediately immersed in RNA stabilization solution. Pools of three membranes from PVR (1 woman and 2 men; age: 49 ± 11, *n* = 3) and of six membranes from primary ERM (3 women and 3 men; age: 73 ± 6, *n* = 6) were prepared for RNA extraction. RNA extraction, RNA concentration determination, and cDNA synthesis were carried out as previously described [34].

### 2.3. Primer Design and RT-qPCR

Primers were designed as previously described [34] to ensure optimal DNA polymerization efficiency and amplification specificity, as well as optimal amplicon length (100 bp to 180 bp). Primer pair efficiency and capability to amplify genomic DNA were determined as previously described [34]. The RT-qPCR reaction was performed as previously described using 2.5 ng cDNA as template [34]. Amplification curves and final amplification melt curve were controlled using the StepOne Software version 2.1 (Applied Biosytems, Carlsbad, CA, USA) and were checked for proper exponential amplification and single *T*_*m*_ peak in the final melt curve after the 40 cycles amplification profile. All nontarget negative controls were performed using Molecular Biology Grade Water—RNase/DNase-free water instead of cDNA.

### 2.4. Double Immunofluorescent Labelling on Membranes from PVR and ERM

PVR and ERM membranes were fixed in 4% buffered formaldehyde and paraffin-embedded and then 3 µm-thick sections were cut. PVR and ERM membranes sections were incubated with AQP1 specific primary antibodies (rabbit polyclonal affinity purified anti-AQP1, dultion 1 : 500 [35]), and alpha-smooth muscle actin (*α*SMA = Acta2), dilution 1 : 500 (Invitrogen), or a rabbit (Sigma-Aldrich, Saint-Louis, MO, USA) or a mouse monoclonal to glial fibrillary acidic protein (GFAP), dilution 1 : 500 (Millipore, Billerica, MA, USA); overnight at 4°C, followed by incubations with biotinylated anti-rabbit IgG, streptavidin-Cyanin2 (Jackson Immunoresearch, West Grove, PA), and anti-mouse IgG coupled with cyanin3 (Jackson Immunoresearch, West Grove, PA). Cyanin2 and cyanin3 are, respectively, green and red fluorochromes as previously described [35]. Cell nuclei were stained with Hoechst 33258 (1 : 5000; Sigma-Aldrich, Saint-Louis, MO, USA). Tissue sections were mounted using FluorSave reagent (Calbiochem, Gibbstown, NJ, USA). As negative controls, sections were incubated with the secondary antibody alone. Images were captured using an Axiocam MRB fluorescent microscope (Zeiss, Göttingen, Germany) running AxioVision digital image processing software.

### 2.5. Labelling Quantification and Statistic Analysis

The number of total cells (DAPI-fluorescent cells) and the number of labelled cells were counted by two independent investigators in areas of 0.32 mm × 0.45 mm (×20 magnification) and 0.65 mm × 0.9 mm (×10 magnification) using both tissue sections labelled with AQP1 and GFAP or with AQP1 and *α*SMA. The data are expressed as percentage of total cell number (DAPI-labelled cells) (mean ± S.E.M.). Spearman correlation coefficients were computed to assess the relation between the variables investigated. Paired Student's *t*-test was used to assess the significant difference between the number of cells labelled with *α*SMA and GFAP and the number of cells labelled with AQP1/GFAP and AQP1/*α*SMA. A *P* value < 0.05 is considered as statistically significant. Data were analyzed using the Statistical Package for the Social Sciences (IBM-SPSS Inc., Chicago, USA).

## 3. Results

### 3.1. mRNA Expression of AQP1 in Membranes from PVR and ERM

**Figure 1:**
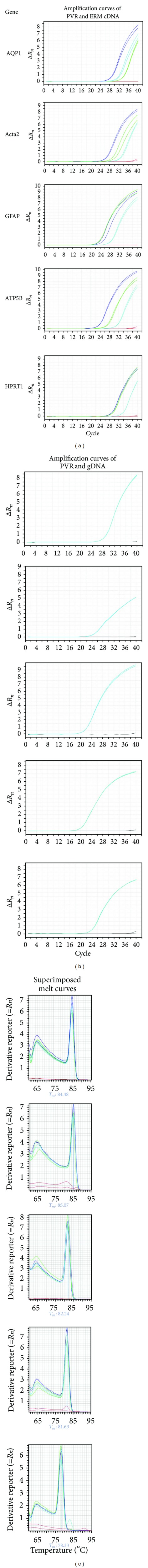
AQP1 mRNA expression in membranes from PVR and ERM. First Column: cDNAs from pools of 3 PVR membranes or 6 ERM membranes were used for RT-qPCR. The amplification curves obtained with cDNA from a positive control (mallow curve), a negative control (red curve), PVR (green curve), and ERM (light blue curve) are shown for each tested gene. Second Column: RT-qPCR amplification curves obtained with cDNA from a positive control (light blue curve) or 2.5 ng genomic DNA (gDNA) (black curve) are shown for each tested gene primers. Third Column: RT-qPCR melt curves obtained following 40 RT-qPCR cycles and with cDNA from a positive control (mallow curve), PVR (green curve), and ERM (light blue curve) or genomic DNA (red curve) are shown for each tested gene.

Gene symbols, gene names, accession numbers, primers sequences, amplicon sizes, and primer efficiencies of the tested genes are described in Table 2; potential genomic DNA amplification using the primer pairs was also tested (data not shown). The genes tested included the genes of interest: AQP1, Acta2 (or *α*SMA), and GFAP, as well as two reference genes: ATP5B and HPRT1. All primers used to amplify these genes had efficiencies comprised between 96 and 107% and did not amplify genomic DNA in the RT-qPCR reaction protocol used [34] (Table 2 and Figure 1, second column).

Both membranes from PVR and ERM pools displayed significant expression of AQP1, Acta2 (*α*SMA), and GFAP mRNAs, as well as of ATP5B and HPRT1 mRNAs (Figure 1, second column). RT-qPCR amplification curves showed significant quantification cycle (Cq) values for the tested genes in both membranes from PVR and ERM pools (Table 3).

Cq values obtained for the positive control are indicated in Table 3 and in Figure 1, first column. Positive control cDNA (+ Ctrl cDNA) used liver cDNA for AQP1 and HPRT1, brain cDNA for GFAP and ATP5B, and kidney cDNA for Acta2 (*α*SMA). RT-qPCR amplification showed typical exponential behaviour for all the tested genes using positive control cDNA or using PVR and ERM membranes cDNA (Figure 1, first column). As expected, all nontarget negative controls did not yield signal amplification. Due to the low amount of cells in membranes from PVR and ERM, samples were pooled. Nevertheless, it was technically impossible to test the purified RNA for possible genomic DNA contamination. Nonetheless, the RNAs were safe for use in RT-qPCR as all primers used did not amplify purified genomic DNA tested in control RT-qPCR reactions (Figure 1, second column). Furthermore, careful analysis of the superimposed RT-qPCR melt curves obtained following 40 q-PCR cycles of the positive control cDNA, PVR cDNA, and ERM cDNA confirmed a single amplicon *T*_*m*_ peak and the absence of a genomic DNA contribution. The amplified signals were consequently considered to be specific for all the tested genes. Therefore, our results unambiguously authenticate the expression of AQP1, Acta2 (or *α*SMA), and GFAP mRNAs in both membranes from PVR and ERM.

### 3.2. Immunofluorescent Detection of AQP1 Protein in Membranes from PVR and ERM

**Figure 2:**
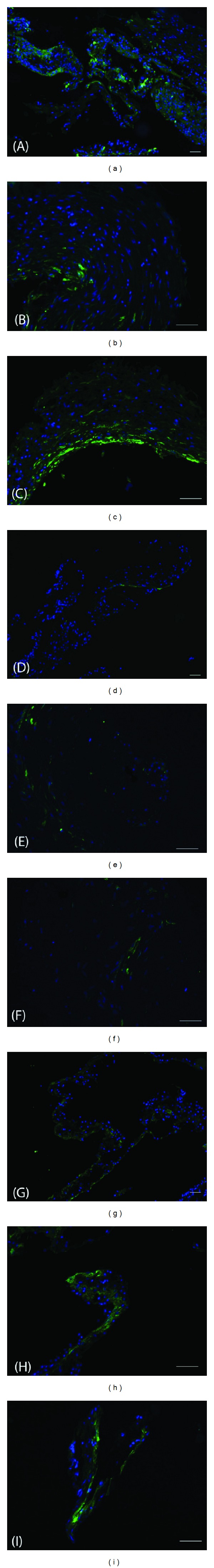
AQP1 expression in membranes from PVR and ERM. Images presented in each row represent different areas of the same membrane. (a–c) PVR number 1, (d–f) PVR number 2, and (e–g) ERM. AQP1 was immunolabelled in green, while cell nuclei were stained in blue with DAPI. Scale bars represent 50 µm.

**Figure 3:**
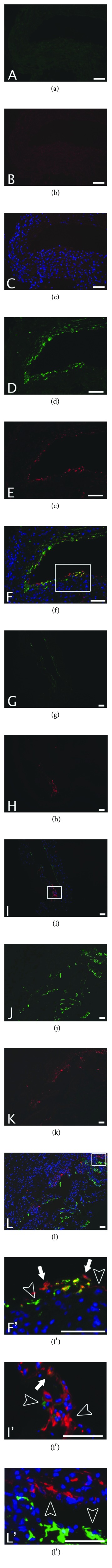
AQP1 expression colocalized with *α*SMA and GFAP. First row of images represents the negative control (a–c), respectively, in green, red, and blue (DAPI stains the cell nuclei). (d) AQP1 expression in green; (e) *α*SMA expression in red; (f) AQP1 and *α*SMA colocalisation (merge of (d) and (f) images) and DAPI; (g) AQP1 expression in green; (h) GFAP expression in red; (i) AQP1 and GFAP colocalisation (merge of (g) and (h) images) and DAPI; (j) GFAP expression in green; (k) *α*SMA expression in red; (l) lack of GFAP and *α*SMA colocalisation (merge of (j) and (k) images) and DAPI. (f′), (i′), and (l′) represent a high magnification of the white square, respectively, drawn in the (f), (i), and (l) picture. Empty arrowheads designate single marker (AQP1 or *α*SMA or GFAP), while solid arrow point out coexpression of AQP1/*α*SMA (f′) or AQP1/GFAP (i′). There was no colocalisation between GFAP and *α*SMA in the (l′) picture. Empty arrowheads designate cells labelled by a single marker, either GFAP in green or *α*SMA in red (l′). Scale bars represent 50 µm.

The expression of AQP1, GFAP, and *α*SMA was next investigated by immunofluorescence on 10 membranes: 5 PVR and 5 ERM. No immunofluorescent staining was detected in the negative controls (Figure 3, first line). All membranes (both PVR and ERM) displayed AQP1 labelling. AQP1 expression was heterogeneous amongst and between the membranes from PVR and ERM (Figure 2). AQP1 immunoexpression was observed throughout the PVR and ERM membranes, with some variability related to the section of membranes studied. AQP1 was mostly expressed on the edges of the membranes (Figures 2 and 3).

**Figure 4:**
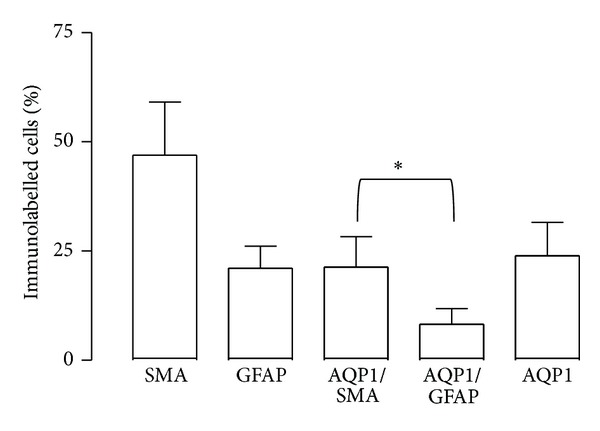
Quantification of cells expressing AQP1, *α*SMA, AQP1/*α*SMA, GFAP, and AQP1/GFAP in PVR and ERM membranes (data are expressed as % of total cell number (DAPI-labelled cells), mean ± S.E.M.). Paired Student's *t*-test assessed the significant difference between the number of cells labelled with AQP1/*α*SMA and AQP1/GFAP (*P < 0.05).

In PVR and ERM membranes, the percentage of cells labelled for AQP1, *α*SMA, and GFAP was 23.89 ± 5.43%, 46.96 ± 12.20%, and 21.06 ± 5.06%, respectively, (Figure 4). In PVR and ERM membranes, the percentage of cells colabelled with AQP1/*α*SMA was higher than the percentage of cells colabelled with AQP1/GFAP (21.32 ± 6.98% versus 8.20 ± 3.61%, *P* = 0.032) (Figure 4).

In PVR and ERM, cells coexpressed AQP1/*α*SMA (Figures 3(d), 3(e), and 3(f)) or AQP1/GFAP (Figures 3(g), 3(h), and 3(i)). Interestingly, *α*SMA and GFAP were expressed by distinct cell types, as no colocalisation could be found between these two markers (Figures 3(j), 3(k), and 3(l)). Image analysis at higher magnification allowed clearly to discriminate between cells expressing only one marker (empty arrow head), with respect to those coexpressing AQP1/*α*SMA (Figure 3(f′)) or AQP1/GFAP (Figure 3(i′)) (solid arrows) and those not coexpressing *α*SMA and GFAP (Figure 3(l′)) (empty arrow head).

**Figure 5:**
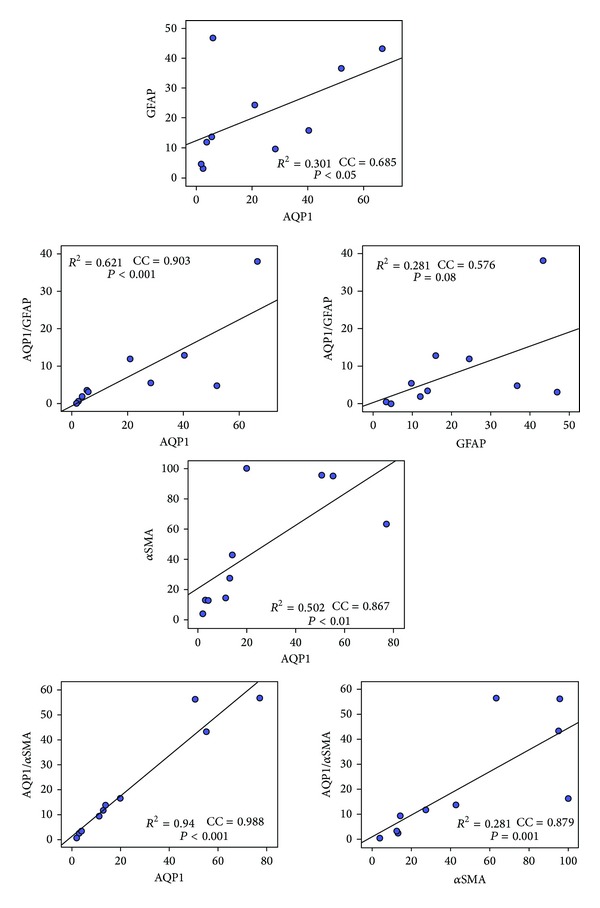
Correlation analysis of cells expressing AQP1, *α*SMA, and GFAP. All the cells labelled with DAPI, AQP1, *α*SMA, and GFAP, with two labels (AQP1/GFAP or AQP1/*α*SMA), were counted and expressed as mean ± S.E.M. (*n* = 10). All the combinations between the variables explored are shown in each graph, where dots represent the mean numbers of cells labelled in each membrane for the investigated variable. Straight lines on each graph represent the best fit linear regression and do not reflect Spearman rank correlation. *R* ^2^ indicates the coefficient of determination, CC is the Spearman correlation coefficient, and *P* is the statistical level of significance. Spearman correlation tests were performed using IBM-SPSS statistical software.

Statistical analysis (using Spearman correlation test) highlighted that the percentages of cells labelled only by AQP1, GFAP, or *α*SMA were positively correlated to each other (Figure 5). The percentage of AQP1-labelled cells was significantly correlated to all the variables explored (Figure 5, first column). Indeed, the percentage of cells expressing AQP1 correlated significantly with that of cells expressing GFAP (CC = 0.685, *P* = 0.029), AQP1/GFAP (CC = 0.903, *P* < 0.001), *α*SMA (CC = 0.867, *P* = 0.001), and AQP1/*α*SMA (0.988, *P* < 0.001) (Figure 5, first column). The percentage of cells expressing GFAP was positively correlated with cells coexpressing AQP1/GFAP (CC = 0.576, *P* = 0.082) but not significantly (Figure 5, second column). The percentage of cells expressing *α*SMA correlated significantly with cells coexpressing AQP1/*α*SMA (CC = 0.879, *P* < 0.001).

## 4. Discussion

In addition to its involvement in transcellular water transport, AQP1 water channel has recently been shown to be involved in cell migration and proliferation [20, 22–27, 36]. Membranes from ERM and PVR appear to be mainly formed as a result of RPE and glial cells that undergo proliferation and migrate onto the surfaces of the retina, although other cell types, such as inflammatory and immune cells, may contribute to the cell proliferation [16, 37]. Prior to exploring if AQP1 could play a role in ERM and PVR development, we first needed to verify AQP1 expression at both the mRNA and protein levels.

In our study, we used membranes from both ERM and PVR. Although ERM and PVR are two different heterogeneous diseases, they share common characteristics such as proliferation, contractility, or cell population types [37–41]. Due to recent changes in surgical technical procedures, we modified our routine vitrectomy from a 20-gauge to a 23-gauge surgery. The membranes peeled with 23-gauge instruments break easily and come in several small pieces making PVR and ERM membranes even more valuable as they contain very limited amounts of cells. Due to the common pathological characteristics and size limitations of the PVR and ERM samples, some PVR and ERM membranes were pooled in order to obtain reliable qPCR data.

The data herein demonstrate for the first time that both membranes from PVR and ERM express AQP1. Indeed, AQP1 mRNA was detected by RT-qPCR and AQP1 protein was detected by immunofluorescence. Furthermore, AQP1 protein expression was expressed heterogeneously amongst and between membranes from PVR and ERM (Figure 1). Heterogeneous expression of proteins is rather common, as recently illustrated for GFAP expression in epiretinal membranes from different pathological conditions [37]. Interestingly, preferential distribution of AQP1 was observed at the edge of PVR and ERM membranes and colocalized with either *α*SMA or GFAP. As cells at the edges of the membranes are usually recognized as being the cells involved in the proliferation and/or migration front, our latter observation may support the fact that AQP1 is indeed involved in cell proliferation and migration during ERM and PVR formation [25, 42, 43]. In accordance with a previous study performed on epiretinal membranes [44], there was no coexpression between *α*SMA and GFAP. Therefore, AQP1 is likely to be expressed by at least two distinct cell types, likely myofibroblastes for AQP1/*α*SMA colabelled cells and glial cells for AQP1/GFAP colabelled cells. Moreover, the percentage of cells labelled with AQP1/*α*SMA was significantly higher than that of cells labelled with AQP1/GFAP. Our data are in agreement with those showing higher number of cells labelled with *α*SMA than with GFAP in an experimental model of PVR [45].

During PVR, RPE undergo a dedifferentiation process and consequently lose their differentiation markers, such as RPE65 and ZO-1, and acquire dedifferentiation markers, such as *α*SMA and ZEB1 [46]. Therefore, we can speculate that the cells expressing AQP1/*α*SMA are dedifferentiated RPE, while the cells expressing AQP1/GFAP are glial cells, possibly Müller cells or astrocytes.

Despite the controversial AQP1 expression by RPE cells [33, 35, 47], the role of AQP1 in cell migration and proliferation has been well described [20, 22–27, 36, 48–50]. Therefore, it is tempting to hypothesise that AQP1 might indeed be involved in the development of both PVR and ERM, especially that preferential AQP1 expression was mostly observed at the edges of PVR and ERM samples. This hypothesis is supported by previous data showing the AQP1-dependent cell migration and AQP-facilitated water influx into dynamic cellular protrusions at the leading edge of endothelial cells [20]. Besides, the heterogeneous expression of AQP1 protein amongst and between PVR and ERM might be related to the proliferation status of the cells present in PVR and ERM. Nonetheless, statistical analysis broadly supports the impressions of the two independent investigators that suggest that the more the membranes express GFAP or *α*SMA, the more the membrane will express AQP1 and vice-versa. AQP1 expression could unfortunately not be correlated with clinical findings, as clinical grading is not easy and not well defined and not systematically done prior to surgery.

In conclusion, the data presented in this paper demonstrate for the first time the expression of AQP1, at both the mRNA and protein levels, in membranes from PVR and ERM. Cell types expressing AQP1 might be dedifferentiated RPE and glial cells as AQP1 positive cells expressed *α*SMA or GFAP. AQP1 is mostly expressed at the edges of PVR and ERM samples. These findings therefore suggest that AQP1 might indeed play a role in the cell migration and proliferation processes occurring during the development of PVR and ERM. These findings warrant further functional investigations aiming at elucidating the potential role of AQP1 in cell migration and proliferation occurring during the development of PVR and ERM.

## Figures and Tables

**Table 1:** Clinical data related to PVR and ERM membranes.

Patient number	Age	Sex	Membrane type	Analysis	Previous use of silicone oil
1	44	M	PVR II	IF	Yes
2	46	M	PVR II	IF	Yes
3	19	M	PVR II	IF	Yes
4	32	M	ERM II	IF	Yes
5	64	M	ERM II	IF	No
6	65	M	PVR II	IF	Yes
7	68	M	ERM I	IF	Yes
8	73	F	ERM	IF	No
9	58	F	PVR II	IF	Yes
10	74	M	ERM I	IF	No
11	63	F	PVR II^#^	RT-qPCR	Yes
14	51	M	PVR II^#^	RT-qPCR	No
12	26	M	PVR I^#^	RT-qPCR	No
13	80	F	ERM I*	RT-qPCR	No
15	76	M	ERM I*	RT-qPCR	No
16	78	F	ERM I*	RT-qPCR	No
17	45	F	ERM I*	RT-qPCR	No
18	65	F	ERM I*	RT-qPCR	No
19	88	M	ERM I*	RT-qPCR	No

PVR I: no previous surgery; PVR II: patients with previous surgery; ERM I: idiopathic; ERM II: other conditions associated such as diabetic retinopathy or PVR or previous surgery; M: male; F: Female; IF: immunofluorescence; RT-qPCR: real-time quantitative polymerase chain reaction; ^#^PVR pooled together to perform RT-qPCR; *idiopathic ERM pooled together. Age is expressed in years.

**Table 2:** Gene symbols, gene name, accession number, primers sequences, amplicon sizes, and primer efficiencies of the tested genes (mean ± S.E.M., *n* = 3).

Gene symbols	Gene name	Accession number	Primer sequences	Amplicon size (bp)	Primer efficiency (%)
hsAQP1	Aquaporin-1	NM_198098	F: 5′-TGGACACCTCCTGGCTATTG-3′	164	107 ± 3
			R: 5′-GGGCCAGGATGAAGTCGTAG-3′		
hsActa2	Smooth muscle actin	NM_001141945.1	F: 5′-GCTATGTGTGAAGAAGAGG-3′	171	97 ± 1
			R: 5′-CACGTAGCTGTCTTTTTGT-3′		
hsGFAP	Glial fibrillary acidic protein	NM_002055.4	F: 5′-GTCAGAAGGCCACCTCAAGA-3′	109	96 ± 2
			R: 5′-CCTGCCTCACATCACATCCT-3′		
hsATP5B	ATP synthase subunit beta	NM_001686.3	F: 5′-AGAGGTCCCATCAAAACCAAAC-3′	152	101 ± 2
			R: 5′-AAAAGCCCAATTTTGCCACC-3′		
hsHPRT1	Hypoxanthine phosphoribosyltransferase 1	NM_000194.2	F: 5′-TGGCGTCGTGATTAGTGATG-3′	137	98 ± 1
			R: 5′-CTCGAGCAAGACGTTCAGTC-3′		

**Table 3:** RT-qPCR Cq values of the tested genes in both PVR and ERM pools. Quantification cycles (Cq) were determined in duplicate in both PVR and ERM pools of samples. Data are expressed as the mean ± S.E.M. of the duplicate measures.

Gene symbol	Cq values		
	PVR	ERM	+ Ctrl cDNA
hsAQP1	30.64 ± 0.02	28.66 ± 0.25	25.72 ± 0.16
hsActa2	27.06 ± 0.53	30.10 ± 0.19	23.91 ± 0.11
hsGFAP	20.81 ± 0.03	25.83 ± 0.07	21.80 ± 0.65
hsATP5B	24.64 ± 0.07	28.78 ± 0.04	21.04 ± 0.13
hsHPRT1	26.84 ± 0.06	31.29 ± 0.04	26.45 ± 0.04
